# Adenoid facies: a long-term vicious cycle of mouth breathing, adenoid hypertrophy, and atypical craniofacial development

**DOI:** 10.3389/fpubh.2024.1494517

**Published:** 2024-12-12

**Authors:** Jiaqi Zhang, Yongwei Fu, Lei Wang, Geng Wu

**Affiliations:** ^1^The First People’s Hospital of Lianyungang, Lianyungang, China; ^2^The Affiliated Lianyungang Hospital of Xuzhou Medical University and The First People’s Hospital of Lianyungang, Lianyungang, China; ^3^The First Affiliated Hospital of Kangda College of Nanjing Medical University and The First People’s Hospital of Lianyungang, Lianyungang, China; ^4^Lianyungang Clinical College of Nanjing Medical University and The First People’s Hospital of Lianyungang, Lianyungang, China

**Keywords:** adenoid facies, mouth breathing, adenoid hypertrophy, malocclusion, craniofacial development

## Abstract

Adenoid hypertrophy (AH) is characterized by pathological hyperplasia of the nasopharyngeal tonsils, a component of Waldryer’s ring, which represents the first immune defense of the upper respiratory tract. The pathogenic factors contributing to AH remain to be comprehensively investigated to date. Although some studies suggest that environmental exposure to smoke and allergens, respiratory tract infections, and hormonal influences likely contribute to the development of AH, further research is necessary for fully elucidating the effects of these factors on the onset and progression of AH. AH is the most common cause of airway obstruction in the pediatric population, with a prevalence rate of 49.7%, and is frequently accompanied by various comorbidities. These patients often present with distinctive dental characteristics, including increased overjet, posterior crossbite, a high palatal plane, narrow dental arches, and facial features characterized by disproportionate alterations in facial height, commonly referred to as “adenoid facies.” Individuals with adenoid facies frequently display abnormal breathing patterns, especially mouth breathing. The present review summarizes the findings of research articles sourced from PubMed, IEEE, and Web of Science over the last 20 years up to September 2024. Several high-quality studies screened using the PICOPS framework reported that perioral muscle dysfunction, dental and skeletal malocclusions, and upper airway obstruction caused by AH are interdependent issues and mutually exacerbate one another. The review summarizes the potential associations and mechanisms linking AH, mouth breathing, and the subsequent development of adenoid facies in children.

## 1 Introduction

Adenoid hypertrophy (AH) is a common condition in pediatric populations that is characterized by a range of respiratory symptoms, including nocturnal snoring, nasal obstruction, mouth breathing, and reduced olfactory sensitivity (1). Apart from these issues, these symptoms of AH contribute to the development of serious secondary complications, including recurrent otitis media (2), obstructive sleep apnea syndrome (3), and sinusitis (4). These complications extend beyond immediate respiratory issues, and can potentially affect normal craniofacial development, neurological functions, and overall health (5). Among these manifestations, mouth breathing is especially predominant, and its prevalence is estimated to range from 11 to 56% in children (6). Despite its high incidence, mouth breathing remains under-recognized by both patients and caregivers, which can potentially delay the administration of appropriate interventions.

Emerging evidence highlights that mouth breathing is a key contributor to the atypical craniofacial development observed in children with AH (7) (8). Although it is traditionally regarded that craniofacial morphology is primarily determined by genetic inheritance, contemporary studies indicate that environmental factors, including oral habits (9), such as pacifier sucking (10), atypical swallowing patterns (11), finger sucking (12), and mouth breathing (7), play a significant role in the etiology of malocclusion (13, 14). Notably, children with AH frequently exhibit distinct dental and facial characteristics, including increased dental overjet, posterior crossbite, high palatal planes, narrow maxillary arches, and adenoid facies, characterized by disproportionate alterations in facial height (15). Malocclusion is especially prevalent in this demographic group, with Class II (16, 17) and Class III (15, 18) malocclusions being more frequently documented.

This review synthesizes the findings of current research on the bidirectional and potentially self-perpetuating relationship between mouth breathing and malocclusion in patients with AH. By integrating the observations of recent studies, the review elucidates the mechanisms by which these conditions reinforce each other in a “vicious cycle” that exacerbates craniofacial anomalies and dental misalignments. The study further aims to provide orthodontists and pediatric dentists with deeper theoretical insights into the mechanisms underlying the development of adenoid facies. The review investigates the factors contributing to this distinct craniofacial presentation to enhance diagnostic precision and ensure the implementation of comprehensive, multidisciplinary, and sequential treatment protocols in clinical practice.

## 2 Methodology

A comprehensive and systematic review was conducted using a structured search strategy across several databases, including PubMed, IEEE, and Web of Science. Database search was conducted using specific keywords and Medical Subject Headings (MeSH) terms, including “adenoid facies,” “mouth breathing,” “adenoid hypertrophy,” “malocclusion,” and “craniofacial development.”

Inclusion criteria were defined for prioritizing the peer-reviewed studies that examined the relationships among AH, mouth breathing, and malocclusion in pediatric populations. The articles published within the last 20 years were prioritized for capturing the recent advancements in the field. The exclusion criteria encompassed articles not available in English as well as case reports.

The articles that met the selection criteria were reviewed for relevance and quality using the PICOPS framework, and the data were extracted and analyzed according to established guidelines. This approach enables the rigorous synthesis of current evidence, promotes transparency, and ensures the reproducibility of findings for future researchers.

## 3 Craniofacial anatomy and risk factors of AH

**Figure 1:**
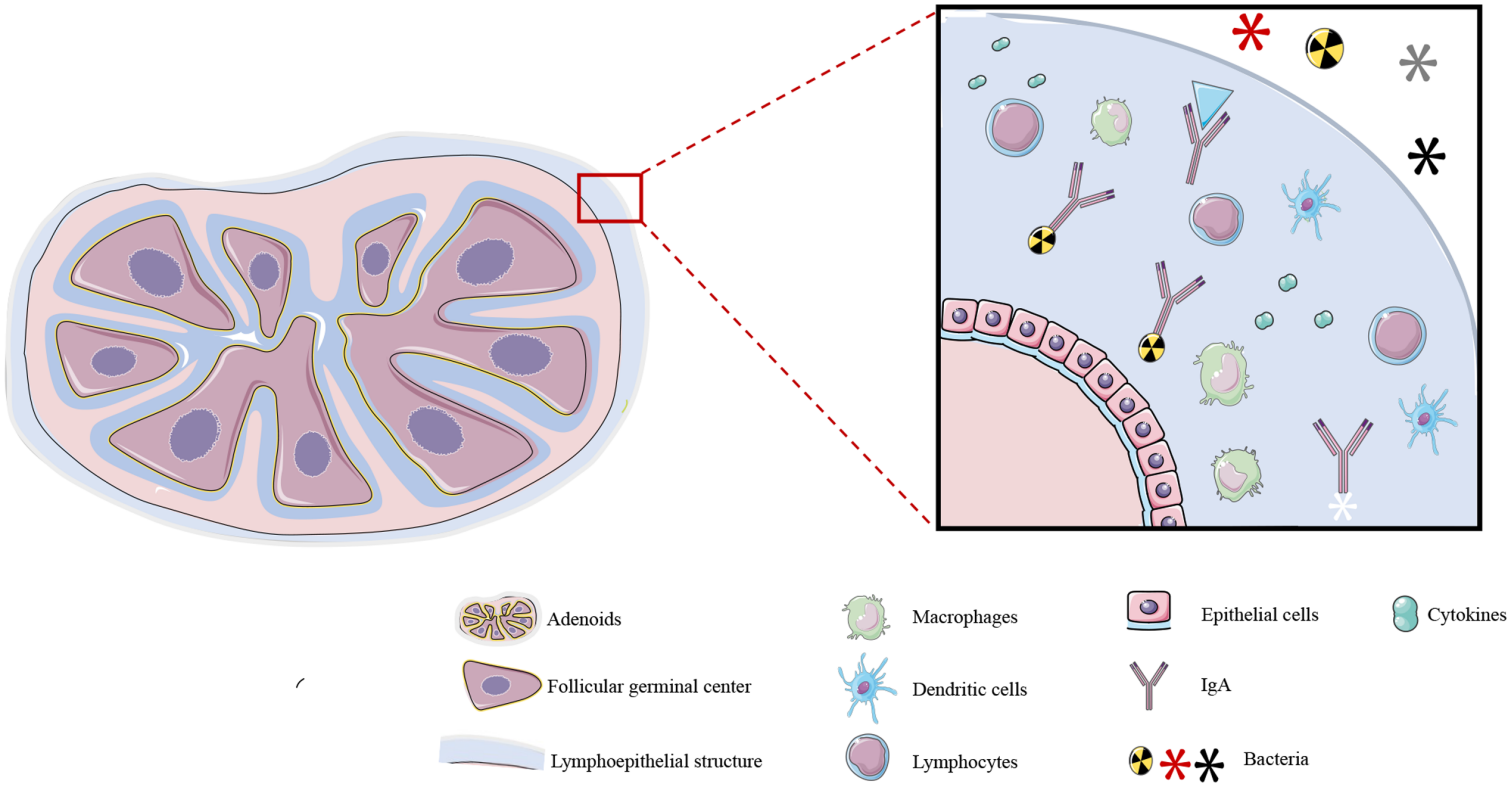
Structure and function of adenoids. Adenoids consist of an inner T lymphocyte-rich follicular germinal center and an interfollicular region, and are covered by a lymphoepithelium. This specialized structure consists of a large number of immune cells, including macrophages, lymphocytes, dendritic cells, and other cell types, and it also secretes cytokines, IgA, and other substances. These structures confer local immunity against microorganisms in the upper respiratory tract. *The illustration was prepared by integrating the findings of existing research. The differences in interpretation may arise due to variations among the conclusions drawn from different research studies.

The adenoids, palatine tonsils, and lingual tonsils form Waldeyer’s ring (19), a component of the lymphoid tissue associated with the upper respiratory system, and collectively regulate immune function in the upper respiratory tract (20). Adenoids are encompassed within a specialized lymphoepithelial structure (21) comprising epithelial cells, lymphocytes, macrophages, and dendritic cells. The lymphoepithelium causes the adenoids to be covered in a thick secretion that attracts and binds microorganisms to confer local immunity (Figure 1) (22). The internal structure of adenoids consists of a follicular germinal center and an interfollicular region, which is formed by the aggregation of T lymphocytes. Adenoids secrete large quantities of secretory immunoglobulin A (IgA) antibody that binds to bacteria and inhibits bacterial colonization in the mucosal epithelium (23). Additionally, the effector T lymphocytes within adenoids can generate effective immune responses by secreting cytokines, chemokines, and bactericidal substances. It is worth noting that adenoids are relatively small in infancy, during which their functions are not apparent. They reach their maximum size at 6–10 years of age, at which point they may occupy a substantial portion of the oral-nasal-pharyngeal space in the retro-palatine region, and their immune functions are most pronounced during this period. However, adenoids shrink in size by puberty and their immune functions correspondingly diminish (24).

AH is the most common obstructive upper airway disorder in children and adolescents worldwide, with a prevalence of 49.7% (25), and respiratory tract infections are the major cause of AH. Human adenovirus is the most frequently detected virus in AH, with a detection rate of 47–71% (26). Other viruses with high detection rates include human enterovirus, rhinovirus, bocavirus, respiratory syncytial virus, and others (27). It has been reported that the smoke produced by the burning of tobacco can increase the risk of upper respiratory tract infections, chronic sinusitis, and chronic otitis media in children (28). A previous study demonstrated that passive exposure to tobacco smoke can significantly increase the production of immunoglobulins by adenoid lymphocytes (29). Allergy and sensitivity to various allergens represent another important risk factor for AH (30). The immune system begins to develop between the ages of 1 and 4 years in children, which consequently increases sensitivity to various antigens during this period, and leads to the successive onset of various allergic diseases, including atopic dermatitis, asthma, and allergic rhinitis (31). In a follow-up study in 2015 involving 1,322 children treated for allergies, researchers conducted skin prick tests for the same allergens on all participants, and observed that children with allergic diseases had a higher frequency of AH than control children without allergic diseases (32). In addition, the hypertrophic surface of adenoids is covered by a biofilm that is rich in microorganisms, environmental pollutants, and food antigens, which further increases the risk of asthma and allergic rhinitis (33). The study by Shin et al. recruited 18 atopic subjects sensitized to more than one common allergen and 22 non-atopic subjects who had undergone adenoidectomy. Subsequent immunoassays conducted using adenoid tissue homogenates revealed that the levels of total IgE and allergen-specific antibodies in the adenoid tissues of children with allergic diseases were significantly higher than those of healthy children without allergic diseases (34). It has been demonstrated that local diseases of the upper respiratory tract, including chronic sinusitis, exudative otitis media, and AH, mutually exacerbate one other. Mucociliary clearance is the most important airway defense mechanism, and infections or inflammation of the adenoids can cause localized epithelial metaplasia and loss of ciliary function in the upper respiratory tract, leading to nasal or middle ear diseases (35). Additionally, chronic infections and inflammation of the respiratory epithelium resulting from nasal or middle ear diseases can induce AH and enhance the secretion of inflammatory mediators (22).

## 4 AH-related mouth breathing promotes dysfunction of perioral muscles

The outer surface of the tooth rests against the labial and buccinator muscles, while the inner surface remains adjacent to the tongue. The opposing forces exerted by these tissues are the primary determinants influencing dental positional stability (36). However, AH-related mouth breathing can lead to atypical tongue positioning, weakening of the orbicularis oris muscle, and overactivity of the buccinator, digastric, mental, and masticatory muscles, leading to malocclusion.

### 4.1 Atypical tongue positioning

**Figure 2:**
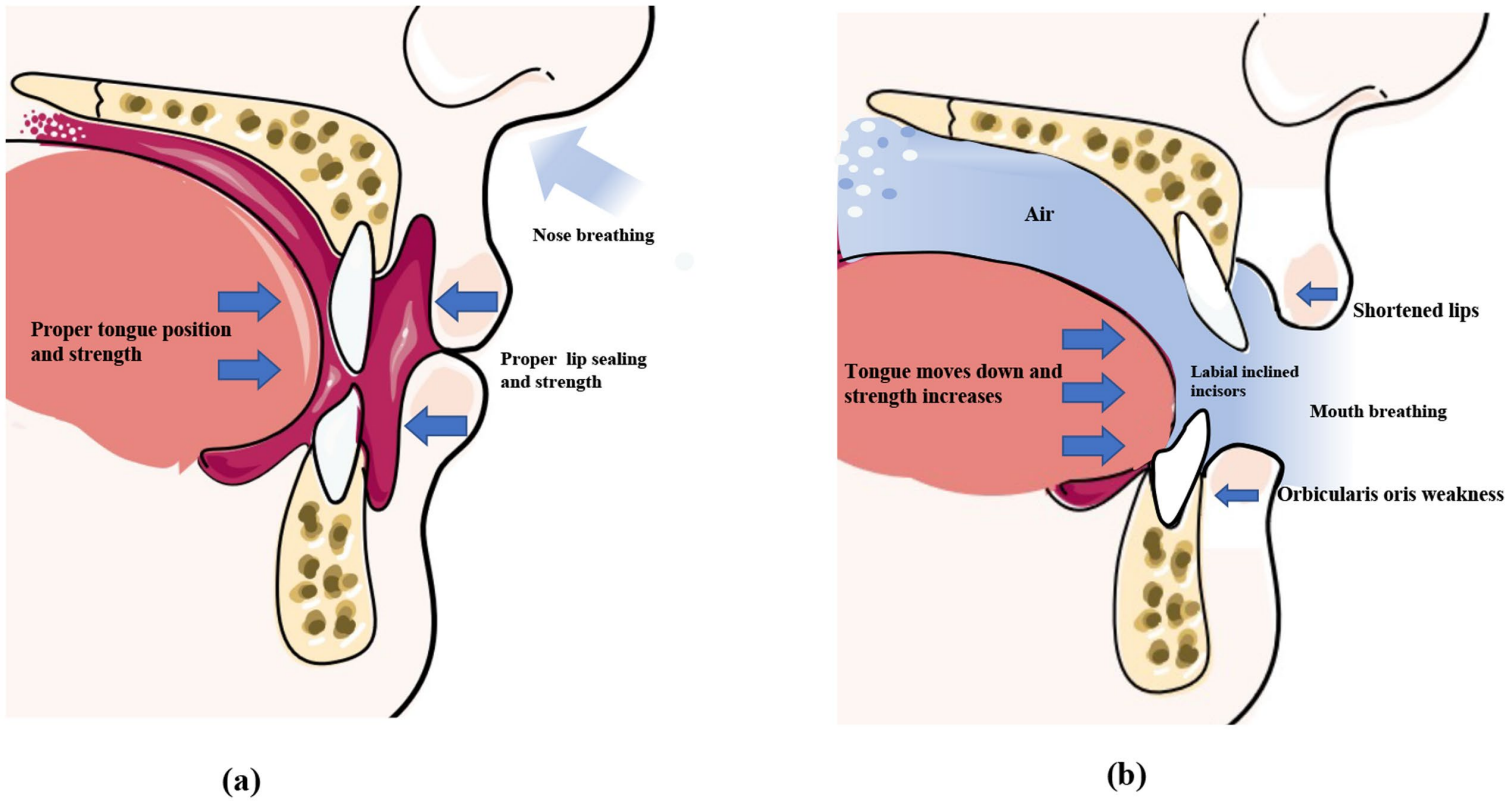
Normal perioral muscle function and the imbalance caused by mouth breathing. Under normal circumstances (left), the lips remain naturally closed at rest, the tongue fills the entire mouth, the forces exerted by the lip and tongue muscles are balanced, and the front teeth remain upright. During mouth breathing (right), the lip seal is insufficient, and the upper lip curls and shortens. The tongue tends to sink, disrupting the balance of perioral muscle strength, which causes the incisors to tilt. *This original illustration depicts the commonly observed clinical patterns. However, individual variations may occur depending on specific cases.

The tongue plays an important role in oral and maxillofacial development, which determines the formation of the dental arch and occlusal relationships (37). The correct positioning of the tongue can be observed in children who breathe through their noses and have proper occlusion (38). The ideal functional position of the tongue is where the lips are lightly closed, the teeth are almost touching, and the tongue is in contact with the palate (39, 40). At rest, the slight force exerted by the tongue is sufficient to move the teeth because the force persists for a prolonged duration (41). There is no absolute balance between the forces exerted by the extraoral and intraoral muscles, which play an important role in the normal positioning of the tongue (42). A cross-sectional study in 2007 compared the effects of mouth opening and closing on lateral cephalometric measurements by fiberoptic nasopharyngoscopy, and the findings revealed that the soft palate moves backward and touches the back wall of the pharynx when breathing through the mouth, thus effectively closing off the nasal cavity. However, during nasal breathing, the base of the tongue moves downwards to reduce the distance between the tongue and the back wall of the pharynx. However, it has been observed that the pressure exerted by the tongue in the pharyngeal region is significantly higher in the supine position than in the upright position when mouth breathing is practiced (Figure 2) (43). Additionally, the position of the tongue is also affected by the posture during mouth breathing. When breathing through the nose, the position and pressure exerted by the tongue remain stable and do not affect breathing irrespective of whether an individual is in an upright or supine position. However, when mouth breathing is performed, the pressure exerted by the tongue is significantly higher in the supine position than in the upright position (44). This is attributed to the weakening of the genioglossus muscle due to mouth breathing (45), which impairs its ability to prevent the tongue from falling back under the action of gravity. This consequently results in the posterior displacement of the tongue, which increases the pressure exerted by the tongue in the pharyngeal region.

### 4.2 Weakening of the orbicularis oris muscle

Lip incompetence is a common clinical sign observed in individuals who practice mouth breathing, and affects approximately 30% of children aged 3–12 years (46, 47). Previous studies have demonstrated that the force exerted by the lips greatly affects dental alignment (48, 49). Therefore, mouth breathing due to AH leads to the weakening of the orbicularis oris muscle, which results in an imbalance in the perioral muscles (50). Consequently, patients who practice mouth breathing tend to have shorter, curled, and thick lips (Figure 2) (51). Wagaiyu et al. performed a cross-sectional study involving 201 schoolchildren aged 11–14 years and observed that the mouth breathers tended to have more curled upper lips. Additionally, the area of the upper lip that shortens and separates from the lower lip is reduced, which potentially exposes the surface of the front teeth and thereby increases the risk of gingivitis (52). Additionally, incompetent lip seals, dry lips, and halitosis are some of the common clinical manifestations in individuals who practice mouth breathing (53). By performing electromyographic (EMG) and cephalometric analyses of 20 adolescents, a previous study revealed that mouth breathing weakens the orbicularis oris muscle, especially the inferior orbicularis oris muscle (Figure 2) (54). This manifests as an insufficiency in the strength of the orbicularis oris muscle when pronouncing certain syllables, such as the phonemes /b/ and /m/, and requires higher EMG activity (55). Additionally, individuals who practice mouth breathing are more prone to EMG fatigue during lip muscle training (56).

### 4.3 Overactivity of the buccinator, digastric, mental, and masticatory muscles

**Figure 3:**
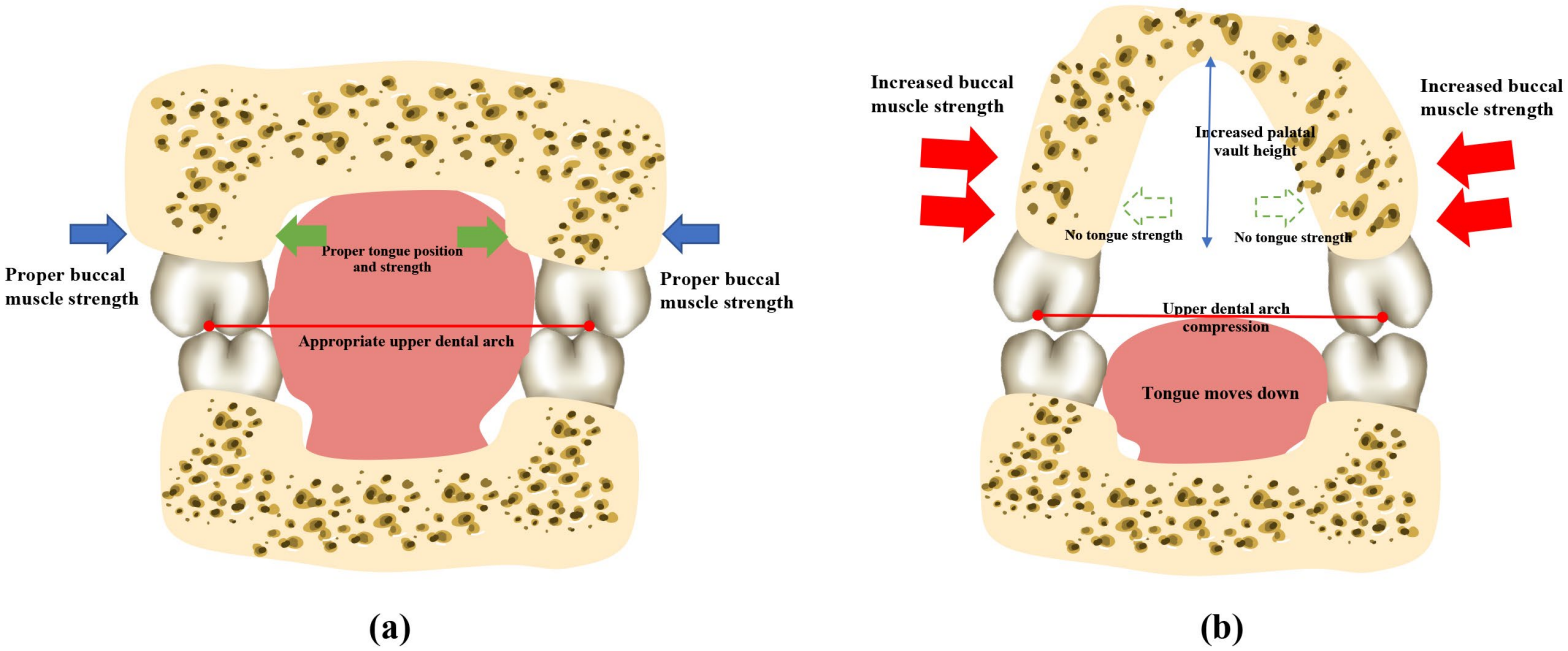
Mouth breathing causes perioral muscle dysfunction and atypical maxillary development. Patients who practice normal nasal respiration generally have balanced perioral muscles, a flat upper palate, and the widths of the upper and lower dental arches are properly coordinated. However, mouth breathing leads to perioral muscle dysfunction, which causes the tongue to sink. The maxilla and the dental arches consequently lose the support provided by the tongue, and the lip strength is weakened due to insufficient lip sealing. The strength of the buccinator muscles is relatively enhanced. The upper dental arch is affected by the imbalanced muscle strength, resulting in a narrow and protruding dental arch, and a higher and sharper palatal fornix. *Note: This original illustration was prepared by integrating the observations of several studies. The variations in the findings may arise due to differences across the various research methodologies and conclusions obtained.

The buccinator muscles, located in the lateral walls of the oral cavity, are responsible for compressing the alveolar bone and increasing the thickness of the cheeks (57). Dysfunction of buccinator muscles can lead to variations in the shape and size of the mandible during growth and development (58). The pressure exerted by the buccal muscles on the alveolar bone increases when the mouth seal is compromised (59), which is likely responsible for the narrow dental arches frequently observed in children who practice mouth breathing (Figure 3). An observational, prospective, multi-center study involving 81 children with a Class II division 1 malocclusion and presenting with one or several functional disorders revealed that the use of muscle function appliances effectively reduces the abnormal tension in the buccal muscles, which can restore the roundness of the dental arches (60).

Another study reported that there is a significant relationship between the activity of the masticatory muscles and facial growth patterns (61). Children with Class II division 1 malocclusion often exhibit abnormal overactivity of the mental, anterior temporal, and masseter muscles (62, 63). By blocking the nasal passages of rhesus monkeys using silicone plugs, a previous study demonstrated that mouth breathing is accompanied by rhythmic hyperactivity of the maxillofacial muscles, including the dorsal tongue, digastric, and levator lip muscles (64). The activation of the masseter and submental muscles can be observed during sleep in mouth breathers, and is possibly attributed to the spontaneous stretching of the muscles to expand the upper airway, which is restricted by mouth breathing. Similarly, the use of oral shields has been shown to reduce the activity of the mentalis, buccinator, and digastric muscles (65).

## 5 AH-related mouth breathing promotes malocclusions

Proper dental alignment and occlusal relationships are essential for oral function, maxillofacial development, and facial esthetics (66, 67). However, the frequency of malocclusions and various occlusal anomalies is significantly higher in children with AH and tonsillar hypertrophy who practice mouth breathing (16, 68, 69).

### 5.1 Class I malocclusion

Crowded dentition is an obvious manifestation of Class I malocclusion in children who practice mouth breathing, and is characterized by narrow dental arches (Table 1). The lateral compression of the upper dental arch is a common occlusal deformity caused by mouth breathing (70, 71), which can be observed in children with deciduous dentition at the age of 2.5 years (72). This is often accompanied by an increase in the depth of the dental arch, which results in an open bite (73). In 2017, a cross-sectional study including 90 children aged 3–12 years with AH and 90 children without AH reported that mouth breathing can also reduce the width of the lower dental arch (74). In addition to the changes in width and depth, the lengths of the upper and lower dental arches were also reduced in mouth breathers aged 3–12 years due to AH (75).

**Table 1:** Relationships between mouth breathing and mismatched dental arches.

Study type^1^	Authors and year of study	Methodology for obtaining measurements	Number of samples	Age (mean or range)	Group design^2^		Observations^4^	References
					Study population	Control group		
Retrospective study	Lysy and Karkazi, 2021	Lateral cephalograms and dental casts	123	9–47 years	57 nose breathers and 19 former mouth breathers who underwent adenoidectomy	47 mouth breathers	Compression of upper dental arch (*p* = 0.00)	(70)
	Shetty, 2021	Cone Beam Computed Tomography (CBCT) scans	200	18–75 years	55 patients with only nasal septal deviation (NSD), 32 patients with only concha bullosa (CB), and 23 patients with both NSD and CB	90 individuals without NSD and CB	Reduction in palatal interalveolar length and increased palatal depth (*p* < 0.001)	(120)
Cross-sectional study	Lione, 2014	3D analysis of digital casts	43	8.5 years (SD 1.6 years)	26 children with allergic rhinitis-induced mouth breathing, skeletal Class I relationship, prepubertal stage of cervical vertebral maturation	17 nose breathers	Reduction in the width of the upper dental arch (*p* < 0.05)	(71)
	Aznar, 2006	Maxillary and mandibular intercanine and intermolar distances measured by oral examination and questionnaires	1,297	3–6 years	Children with bad oral habits: 234 children with dummy habits, 152 children with finger sucking, children with mouth breathing^3^	Children without bad oral habits: 1063 without dummy habits; 1,145 who did not practice finger-sucking; children without mouth breathing	Reduced maxillary intercanine distance (*p* = 0.002)	(74)
	Cheng, 2023	3D facial images measured using the 3dMDFace system (3dMD Inc., United States)	65	10–12 years	35 children with mouth breathing (17 males and 18 females)	30 children with nasal breathing (15 males and 15 females)	Narrowing of the mandibular width (*p* < 0.05)	(121)
	Bakor, 2011	Electromyography, oral examination, and X-ray	30	13.1 years	10 children with mouth breathing and 10 children who underwent tracheotomy	10 children with nasal breathing	Reduction in facial, maxillary, and mandibular width (*p* < 0.05)	(122)
	Diouf, 2019	Dental casts and X-ray	86	9.77 years	42 children with obstructive adenoids and mouth breathing	44 children without obstructive adenoids	Reduction in posterior mandibular length (*p* = 0.04)	(73)
	Osiatuma VI, 2017	Dental casts	180	3 to 12 years	90 children with AH	90 children without AH	Shorter maxillary and mandibular arches (*p* = 0.049)	(75)

1 All the studies included represent empirical research and provide data-driven insights into the relationships among mouth breathing, malocclusion, and adenoid facies.

2 The subgroups characterized by oral breathing have been summarized, while the other subgroups were excluded due to space constraints. Detailed insights into the methodologies and the findings for the different subgroups are available from the corresponding references.

3 The number of mouth breathers was not clearly addressed in literature.

4 Negative outcomes observed among mouth breathers.

### 5.2 Class II malocclusion

Previous studies have demonstrated that Class II malocclusion is the most common occlusal anomaly in children who practice mouth breathing (7, 76, 77). The occlusal presentation of Class II malocclusion is primarily classified into two types, namely, increased anterior dental overjet (Class II division 1), and a deep anterior overbite (Class II division 2) (78, 79), and the former is frequently accompanied by an increased anterior dental overjet. It has been proposed that children who practice mouth breathing present with narrower dental arches and increased anterior dental overjet (Table 2) (15, 80). A previous study reported that the narrowing of the maxillary dental arch is more severe and the upper incisors are more labially inclined in mouth breathers (81).

**Table 2:** AH-related mouth breathing causes dental misalignment.

Study type^1^	Authors and year of study	Methodology for obtaining measurements	Number of samples	Age (mean or range)	Group design^2^		Observations^3^	References
					Study population	Control group		
Follow-up study	Zettergren-Wijk, 2006	Clinical orthodontic examination and lateral cephalograms	34	5.7 years	17 children (10 boys and 7 girls, mean age 5.6 years) diagnosed with Obstructive Sleep Apnea (OSA) syndrome	17 age- and gender-matched children (mean age 5.8 years)	Enhanced posterior inclination of the mandible, increased anterior inclination of the maxilla, increased lower anterior facial height, retroclination of the upper and lower incisors (*p* < 0.05)	(89)
Cross-sectional study	Festa, 2021	ENT examination and clinical orthodontic examination	221	6.2 years (SD 2.5 years)	221 children with tonsillar hypertrophy and mouth breathing	No control group	Increased dental overjet (*p* < 0.05)	(16)
	Diouf, 2019	Dental casts and X-ray	86	9.77 years	42 children with AH and obstructive mouth breathing	44 children with non-obstructive adenoids	Reduction in posterior mandibular length (*p* = 0.04), increased overbite (*p* = 0.04), increased depth of dental arch (*p* = 0.02), increased open bite (*p* = 0.03)	(73)
	Souki, 2009	Clinical orthodontic examination and interview	401	6.5 years (SD: 2.6 years)	401 children with mouth breathing	No control group	Higher prevalence of lateral crossbite (*p* < 0.05)	(86)
Cohort study	Löfstrand-Tideström, 2010	Clinical orthodontic examination, lateral cephalograms, dental casts, and questionnaires	644	4, 6, and 12 years	25 children with sleep-disordered breathing	24 children without sleep-disordered breathing	Reduction in the width of the maxilla, anterior open bite, and lateral crossbite (*p* < 0.05)	(87)
	D’Ascani, 2010	ENT examination, clinical orthodontic examination, and lateral cephalograms	196	8.8 years	98 children (59 males and 39 females; mean age 8.8 years; age range 7–12 years) with obligate mouth breathing secondary to NSD	98 age- and sex-matched control children with nasal breathing	Increased anterior facial height, higher palatal height, enhanced dental overjet, significantly retrognatic position of the maxilla and mandible, and a higher prevalence of crossbite (*p* = 0.02)	(84)

1 All the studies included represent empirical research and provide data-driven insights into the relationships among mouth breathing, malocclusion, and adenoid facies.

2 The subgroups characterized by oral breathing are summarized, while the other subgroups were excluded due to space constraints. Detailed insights into the methodologies and the findings related to the different subgroups are available from the corresponding references.

3 Negative outcomes associated with mouth breathing.

In 2021, otolaryngologists and orthodontists conducted a cross-sectional study to evaluate 356 children with AH and tonsillar hypertrophy who practiced mouth breathing. The findings revealed that 81.4% of the mouth breathers presented with Class II malocclusion, with an increased anterior dental overjet being the most prominent feature (16). It has been reported that the long-term use of oral appliances in patients with sleep apnea due to mouth breathing can lead to objective and significant changes in dental malocclusion, including an improvement in dental overjet, independent of the subjective experiences of the patients (82).

### 5.3 Class III malocclusion

By analyzing the relationship between malocclusion types and respiratory factors in 72 children with and without crossbite during the early mixed dentition phase, a previous study demonstrated that Class III malocclusion is frequently accompanied by ear, nose, and throat (ENT) disorders, which are closely associated with mouth breathing (83). A comparative cephalometric analysis involving 98 children with mouth breathing and 98 children with nasal breathing reported a high prevalence of anterior crossbite and anterior open bite among the mouth breathers (Table 2) (84). Posterior crossbite, including lateral crossbite (85), is also common in children who practice mouth breathing (86, 87). Mechanistically, mouth breathing causes muscular dysfunction, which leads to the forward movement of the tongue, thereby prompting the patient to involuntarily protrude the mandible. This eventually leads to the development of an anterior crossbite, which if not corrected in time, can hinder normal maxillary development, while the mandible may undergo unrestricted overdevelopment and ultimately result in typical skeletal Class III malocclusions. Last but not least, deviations from the intended trajectory of tooth eruption are also frequently observed in children over 3 years of age with mouth breathing (88). Excessive molar eruption has been observed in children with mouth breathing (89), which may lead to a clockwise rotation of the mandible and a disproportional increase in the anterior lower vertical height of the face (90).

## 6 AH-related mouth breathing promotes the atypical development of the facial skeleton

Mouth breathing, often resulting from AH, is associated with the atypical development of the facial skeleton, and particularly affects the mandible, maxilla, and hyoid bone. However, the effects of mouth breathing on maxillofacial bone structure are less pronounced in adults, which could be attributed to the reduced secretion of growth hormone (somatotropin) observed in children with AH who habitually engage in mouth breathing.

### 6.1 Maxilla

Mouth breathing is associated with maxillofacial growth and development, and affects the development of orofacial structures, including maxillary narrowing (85), enhanced facial convexity (91), mandibular retraction, and increased facial height (92). Mouth breathing leads to physiological changes in the upper respiratory tract that can cause adaptive changes in the maxilla (Table 3) (93, 94). An increase in the height of the palatal vault is the most common manifestation of maxillary dysplasia resulting from mouth breathing (71). Patients with mouth breathing due to AH-induced nasal obstruction tend to have a higher and sharper palatal fornix at the canine, premolar, and molar levels (Figure 3) (75). By performing three-dimensional (3D) analyses of digital dental models, previous studies have demonstrated that mouth breathing markedly reduces the total surface area and volume of the palate (74, 95). Additionally, an observational study conducted over a period of 3 years revealed that maxillary growth is slower in children with mouth breathing (74, 96).

**Table 3:** AH-related mouth breathing causes atypical development of the facial skeleton.

Study type^1^	Authors and year of study	Methodology for obtaining measurements	Number of samples	Age (mean or range) years	Group design^2^		Observations^3^	References
					Study population	Control group		
Cross-sectional study	Lione, 2014	3D analysis of digital dental casts	38	8.5 years (SD 1.6 years)	21 children with mouth breathing due to allergic rhinitis, early mixed dentition, skeletal Class I relationship, and pre-pubertal stage of cervical vertebral maturation	17 nose breathers	Significant reduction in palatal surface area and volume	(95)
	Chung Leng Muñoz, 2014	Lateral cephalometric radiographs	118	6–12 years	53 children with mouth breathing	65 children with nose breathing	Enhanced mandibular retrusion, greater inclination of the mandibular and occlusal planes, increased elevation of the hyoid bone (*p* < 0.05)	(17)
	Galeotti, 2019	Lateral radiograph, orthodontic and ENT examinations	47	5.75 years (SD 1.99 years)	47 children with obstructive sleep apnoea	No control group	Hyperdivergent maxillomandibular growth pattern (*p* = 0.007)	(98)
	Chambi-Rocha, 2018	Clinical orthodontic examination, parent questionnaire, and lateral radiographs	98	7–16 years	56 children with mouth breathing	42 children with nose breathing	Enhanced palatal length (*p* = 0.049), increased vertical dimension of the lower anterior face (*p* = 0.015), and downward displacement of the hyoid bone (*p* = 0.017)	(99)
Follow-up study	Niemi, 2019	Clinical orthodontic examination, ENT examinations, and profile photographs	51	2.5–3 years	32 snorers as reported by parents (18 females, 14 males; snoring ≥3 nights/week)	19 non-snorers (14 females, 6 males)	More convex profile (*p* < 0.05)	(91)
Retrospective study	Harari, 2010	Medical history, complete physical examination, and questionnaires	116	10–14 years	55 children with mouth breathing	61 healthy children with nose breathing	Marked backward and downward rotation of the mandible, increased angle of mandibular plane, and higher palatal plane (*p* < 0.05)	(15)
	Mohamed, 2022	CBCT scans	126	7–12 years	66 children with mouth breathing	60 children with nose breathing	Downward displacement of the hyoid bone (*p* < 0.05)	(106)

1 The studies included here represent empirical research and provide data-driven insights into the relationships among mouth breathing, malocclusion, and adenoid facies.

2 The subgroups characterized by oral breathing have been summarized, while the other subgroups were excluded due to space constraints. Detailed insights into the methodologies and the findings related to the different subgroups are available from the corresponding references.

3 Negative outcomes associated with mouth breathing.

### 6.2 Mandible

It has been demonstrated that skeletal anomalies, including increased vertical mandibular growth, correlate with the AH-induced narrowing of the upper nasopharyngeal cavity (97, 98). Children who habitually breathe through their mouths often present with a retruded mandible, increased anterior lower facial height, and a steeper inclination of the mandibular and occlusal planes (Table 3) (99) (17). This facial pattern, often referred to as “long face syndrome” or “high-angle profile,” is characterized by vertical discrepancies in the lower face, which can sometimes improve following adenoidectomy (70). Both Class II and Class III malocclusions are frequently observed in children with mouth breathing, as altered breathing patterns can affect craniofacial growth. However, Class II malocclusions, which are marked by mandibular retrusion, are typically more prevalent in this population (100, 101). Although Class III malocclusions have also been observed in children with mouth breathing, they are proportionally less common than Class II malocclusions. Additionally, the mandibular growth pattern in cases with Class II malocclusions is generally neutral to hypo-divergent in the sagittal plane, indicating that vertical growth tendencies are less pronounced in these patients (102). Additionally, a meta-analysis demonstrated that children with mouth breathing tend to exhibit rotational changes in the mandible and maxilla relative to the cranial base, which further affects craniofacial structure (103).

### 6.3 Hyoid bone

The hyoid bone is a key component of the maxillofacial complex, and its position is also affected by mouth breathing (Table 3) (104). Previous studies have reported varying findings on its position in relation to different breathing modalities. Cephalometric analyses of children aged 7–16 years have shown that the position of the hyoid bone is significantly lower relative to the mandibular plane in mouth breathers compared to nasal breathers (99). Conversely, research on preschool children with airway obstruction suggests that the displacement of the hyoid bone is likely predominantly affected by obstructive conditions instead of breathing habits alone (105). Additionally, cone-beam computed tomography of children aged 10–12 years demonstrated that the hyoid bone adopts a vertically higher and more posterior position in mouth breathers compared to nasal breathers (106). These discrepancies may stem from the differences across the various imaging techniques and developmental factors. However, maintaining the positional stability of the hyoid bone is crucial for ensuring the patency of the nasopharyngeal airway (107). The downward and backward displacement of the hyoid bone associated with mouth breathing may contribute to nasopharyngeal airway stenosis, thereby perpetuating mouth breathing. In summary, the findings obtained from existing literature provide diverse perspectives on the position of the hyoid bone in mouth breathing, which highlights the necessity for further research to elucidate the factors that influence these variations across different populations and methodologies.

### 6.4 Somatotropin secretion

Mouth breathing is one of the major risk factors that affect normal craniofacial development in children. However, it should be noted that mouth breathing has a relatively minor effect on the maxillofacial bones of adult patients (76), which could be related to the inhibition of somatotropin secretion in children with AH who practice mouth breathing.

It has been observed that the serum levels of insulin-like growth factor-1 (IGF-1) (108), IGF binding protein-3 (IGFBP-3), and plasma ghrelin (GH) (109) are significantly lower in children with AH and tonsillar hypertrophy who practice mouth breathing (62, 110). Furthermore, the reduction in the serum levels of IGF-1, IGFBP-3, and GH is associated with reduced appetite and restricted energy intake (111). This suggests that AH affects maxillofacial growth and development in children, and is also associated with the inhibition of growth hormone secretion. A prospective study examining the growth characteristics of children under 5 years of age following adenoidectomy revealed that the linear growth measures, including height and weight, improved postoperatively and correlated with an improvement in the IGF-1/GH ratio (112).

## 7 Mutual exacerbation of malocclusion, AH-related mouth breathing, and muscular dysfunction

**Figure 4:**
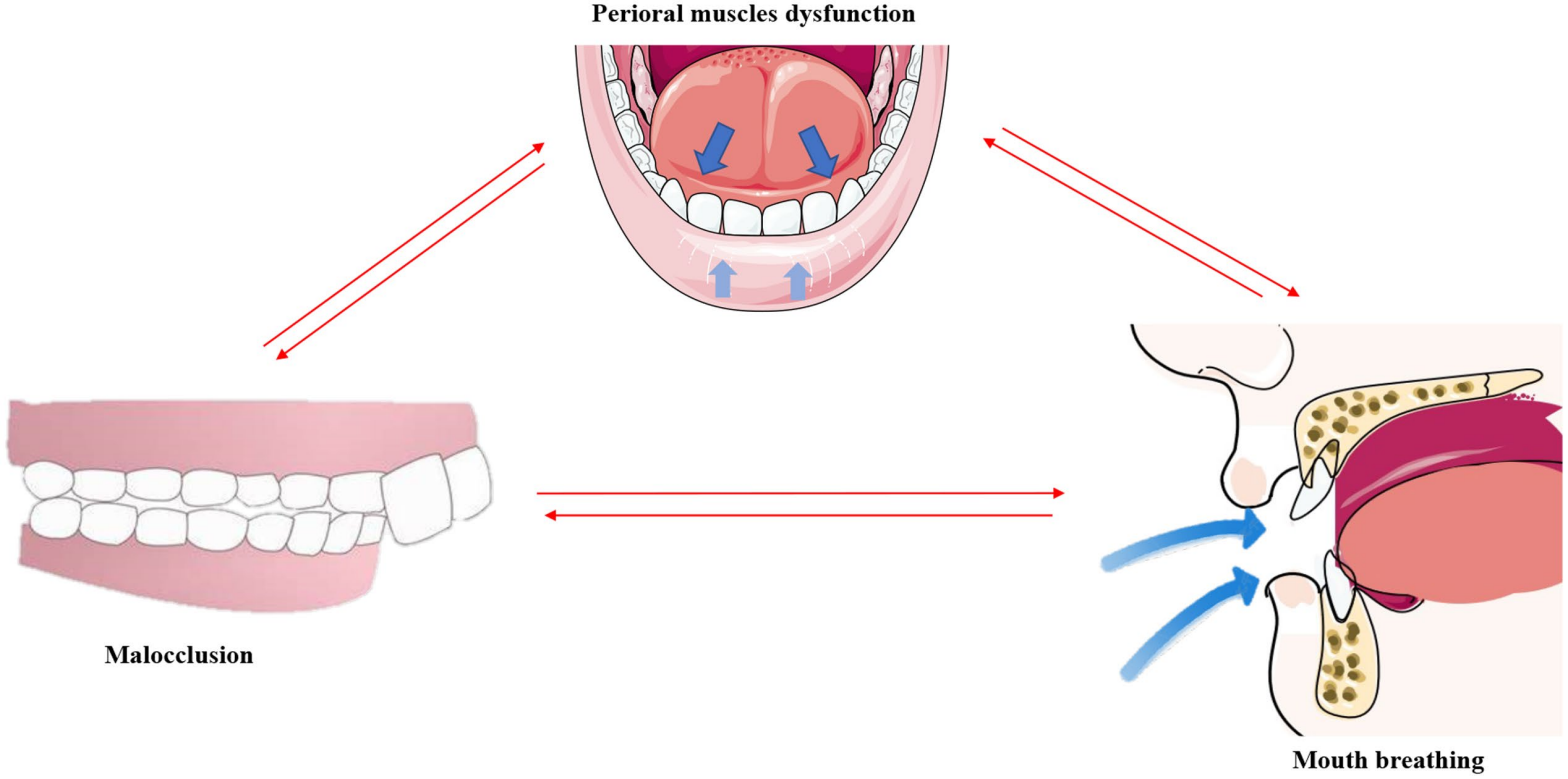
Malocclusion, mouth breathing, and muscular dysfunction mutually exacerbate one another. *This original illustration summarizes the reciprocal influences of these factors; however, future research may present different perspectives and conclusions.

Interestingly, the structural changes in the teeth, facial muscles, and bones caused by mouth breathing do not occur in an independent manner, but are instead closely interconnected (Figure 4).

An imbalance in the perioral muscles leads to dental misalignment, and the concurrent onset of malocclusion further exacerbates the muscular imbalance (113). As aforementioned, mouth breathing causes the tongue to drop and shift posteriorly. By employing cone-beam computed tomography (CBCT), a previous study revealed that the tongue adopts a lower position and has a smaller body in Class II malocclusions (114). The tongue also tends to be in a lower position in the mouth in children with a posterior crossbite (115). This indicates that malocclusion further exacerbates muscular dysfunction as proper dental alignment can help balance the strength between the tongue and the labial and buccal muscles.

It has been reported that mouth breathing and muscular dysfunction exacerbate one other. Comparative evaluation of the EMG activities of the orbicularis oris and mentalis muscles in children with mouth breathing revealed that the EMG activity of the mentalis muscle is higher in patients with mouth breathing (55), indicating dysfunction of the perioral muscles in these individuals (116). The overactivity of the submandibular muscles may in turn exacerbate mandibular retraction and mouth breathing, thus forming a negative feedback loop with regards to muscular function.

It is known that mouth breathing and malocclusion exacerbate one other (8). A comparative study of upper and lower pharyngeal airways of 80 subjects with Class I and Class II malocclusions revealed that the width of the nasopharyngeal cavity is narrower in patients with Class I and Class II malocclusions (117), which indicates an increased likelihood of airway obstruction due to malocclusion. Correspondingly, the use of functional appliances for correcting mandibular retrognathism can decrease upper airway resistance and reduce mouth breathing in adolescents (118). A follow-up study involving 49 prepubertal children with severe obstructive mouth breathing revealed a significant increase in transverse maxillary width and a marked improvement in dental crowding after 1 year of adenoidectomy (119).

## 8 Conclusion

In summary, the occurrence AH in children, caused by the passive inhalation of tobacco smoke, exposure to allergens, or other forms of upper respiratory tract inflammation, can obstruct the nasopharyngeal cavity, leading to mouth breathing. Mouth breathing can cause various functional disorders of the perioral muscles, including the weakening of lip muscles and drooping of the tongue, as well as dental misalignments, such as increased overjet, open bite, crossbite, and narrow dental arches. These factors ultimately contribute to the atypical development of the maxillofacial skeleton, including a higher and sharper palatal fornix, a receding mandible, and a downwardly displaced hyoid bone. AH further inhibits the secretion of somatotropin, which exacerbates maxillofacial skeletal dysplasia in children with mouth breathing. Importantly, perioral muscle dysfunction, malocclusion, and upper airway obstruction caused by hypertrophic adenoids are not independent issues, but are instead closely interconnected and mutually exacerbating, and their combined effects lead to the development of adenoid facies.
